# Optimization of Signal Timing for Urban Expressway Exit Ramp Connecting Intersection

**DOI:** 10.3390/s23156884

**Published:** 2023-08-03

**Authors:** Mingjun Deng, Fei Chen, Yongtai Gong, Xiang Li, Shuhang Li

**Affiliations:** School of Transportation Engineering, East China Jiaotong University, Nanchang 330013, China

**Keywords:** urban traffic, bi-level programming, signal timing optimization, particle swarm optimization, traffic simulation

## Abstract

To alleviate the traffic problems of congestion and queue overflow on a mainline at the intersection of an urban expressway exit ramp articulation during peak hours, a bi-level programming optimization model of signal timing is proposed. The lower-level optimization objective is to maximize the capacity of the expressway exit ramp that articulates with the entrance road, while the upper-level optimization objective is to minimize the average vehicle delay and the number of stops per vehicle, taking into account the queue length in the direction of the ramp and other directions. The particle swarm optimization algorithm is selected to solve the proposed model, applied to a real case, and is validated using MATLAB and VISSIM simulation platforms. The simulation results show that the average vehicle delay and the number of stops per vehicle in the exit ramp on the expressway are reduced by 22.09% and 18.60%, while those in the intersection area are reduced by 20.96% and 17.19%, respectively. The conclusion indicates that the signal timing scheme obtained by this method can effectively improve the traffic efficiency at the intersection of the exit ramp on the expressway and alleviate the problem of congestion and the overflow of the exit ramp back to the mainline.

## 1. Introduction

An urban expressway system is responsible for linking the traffic functions of various functional groups or subdivisions of a city and, through orderly connection with conventional roads, constitutes a fast and convenient travel corridor. However, in recent years, with the dramatic increase in car ownership leading to the deteriorating levels of urban expressway services, vehicle congestion is common; in particular, the situation in which mainline expressway vehicles in the exit ramp area “cannot get out” is gradually intensifying, which causes the total congestion of the mainline. Therefore, how to slow down traffic congestion on an expressway exit ramp, as well as a ground intersection interface area, in order to improve an urban expressway network alongside its interface road traffic level is an urgent issue that needs to be studied.

Due to the interdependence of operational efficiency between expressway segments and connecting roads, there have been numerous literature reports over the past few decades regarding parallel control strategies for both urban expressways and local arterials. However, most of these studies have focused on entrance ramp metering and its coordination. The equally important issue of exit ramp control has not received sufficient attention. During peak hours, queues may form at exit ramps and potentially propagate upstream, affecting the operation of a mainline expressway. Taking into account queuing at right-turn lanes, Newell [1] proposed a model to assess the delay caused when queues from exit ramps spill back onto the mainline expressway.

In response to the issues, many scholars have conducted relevant research. Günther et al. [2] proposed a model that diverts some vehicles to surface streets and which prioritizes the flow of vehicles at an exit ramp. Spiliopoulou et al. [3] developed a real-time route diversion model that, when detecting queue overflow at an exit ramp, activates a control module to divert some exit ramp traffic to alternative routes, aiming to prevent queue overflow at the exit ramp. However, due to the possibility of drivers ignoring diversion instructions, Spiliopoulou et al. further proposed another control model that requires the temporary closure of a ramp to enforce route diversion. They aim to benefit the flow of vehicles at the exit ramp by sacrificing the convenience of surface street users, which may be perceived as unfair to these users.

Apart from the aforementioned methods, a more effective approach to alleviate such queue overflow at exit ramps is to optimize traffic signals connecting exit ramps to local main roads. Kim et al. [4] proposed a signal control model that used linear programming to calculate optimal signal timing and minimize delays on both exit ramps and mainlines, thus addressing congestion issues on urban freeway exit ramps, and their simulation results showed that the model can increase throughputs, reduce delay times, and prevent capacity decreases on a freeway. Yin et al. [5], based on a detailed description of an adaptive and cooperative signal control strategy, presented the specific deployment schemes of various schemes at the entrance of a ramp, an exit side road, and the downstream intersection of an exit. Their simulation results showed that the adaptive and cooperative control strategy can improve road traffic capacity in bottleneck areas and effectively alleviate traffic congestion on urban expressways. Zhang et al. [6] established a bi-level planning model based on genetic algorithm and VISSIM simulation platform to optimize the signal control scheme in an area of an expressway exit ramp connected to an oversaturated intersection to prevent the traffic problem of expressway exit ramp vehicles queuing on the mainline, and their results showed that the model was effective in preventing queue overflow and improving the overall performance of the area. The purpose of the abovementioned studies is to prevent queue overflow. However, they opted to install traffic lights at exit ramps, simplifying the interaction between expressways and regular urban roads [7]. Nevertheless, many Chinese cities do not have traffic lights at the exit ramps of their expressways, making it inappropriate to directly apply these expressway coordination control methods to such urban roads.

Pang et al. [8] proposed an adaptive control method for exit and entrance ramps from the perspective of vehicle–road coordination and verified the effectiveness of combining the method of determining the priority of exit ramp phase correlation with the demand and capacity of exit ramps using a cellular automaton model. Yang et al. [9] developed an integrated control system to prevent the queue overflow of vehicles on an exit ramp, which included three main functions: an estimation of the exit ramp queue, the adaptive signal operation on the main road, and the prioritized control of the expressway exit ramp. Their experimental results showed that the proposed control system can indeed improve overall network performance compared to other operation strategies. Pang et al. [10] proposed an integrated model of adjacent signal intersection coupling by defining the nodes in an expressway exit ramp and the adjacent intersection system, and optimized the signal timing scheme of the intersection by designing a controller based on an urban expressway-integrated coordination control. The simulation experiments verified that the proposed method could effectively suppress traffic congestion. However, one limitation is that they only considered queue length in the direction of exit ramps and did not account for queue length in other entrance lanes at connecting intersections, which may have some negative implications for regional traffic.

Many exit ramp optimization models often solely consider queue length or delay as the sole evaluation criterion, but this does not adequately evaluate the performance of the entire exit ramp and connecting intersection. To comprehensively optimize signal strategies, Liu et al. [11] addressed congestion issues in expressway exit ramp areas by considering factors such as delay time, queue length, and stopping frequency, and proposing a bi-level signal timing optimization model. They employed an improved genetic algorithm to solve the model and tested it with PTV VISSIM software (https://doi.org/10.1007/s13369-020-04835-6, accessed on 29 July 2023). The results show that their model effectively improves traffic efficiency at off-ramp intersections compared with the original planning and single-stage models. On the other hand, Zhang et al. [12] proposed a coordinated control method based on a mixed logical dynamic (MLD) model, utilizing a bi-level planning genetic algorithm to coordinate the control of exit ramps and downstream intersections. They further validated the effectiveness of the bi-level programming model through simulations using VISSIM software.

To achieve a more comprehensive signal timing optimization, this study considered factors like capacity, delay, queue length, and number of stops per vehicle. As heuristic algorithms have gained widespread attention, many researchers have adopted swarm intelligence optimization algorithms to find solutions for their models. Among heuristic algorithms, swarm intelligence optimization algorithms, such as Particle Swarm Optimization (PSO), are favored for their simplicity, fast convergence, and ease of implementation compared to precise algorithms (branch and bound [13], trust region method [14], etc.) and other heuristic algorithms (genetic algorithm [15], evolutionary algorithm [16], etc.) in solving optimization problems. Furthermore, swarm intelligence optimization algorithms have proven to be useful within the framework of existing signal timing models. Xu et al. [17] established a mathematical model with the average vehicle delay, the minimum number of stops, the maximum overall intersection capacity, the effective green time for each phase, and the intersection cycle length as constraints, and they employed the PSO to optimize intersection capacity. Hence, we chose to use the PSO to solve our model.

Based on the existing research findings, the study of congestion issues on exit ramps primarily involves developing coordinated control models to optimize intersection signal parameters at the between exit ramps and connecting intersections. These methods typically focus on granting more green time to the entrance lanes in the direction of the exit ramp to improve the dispersal capacity of the queues on the exit ramp. However, during peak hours, the direction of the exit ramp often experiences oversaturation. Simply increasing the green time for that direction may lead to excessively long red times for other directions, resulting in the overflow of queues to upstream intersections and causing regional traffic congestion.

In light of the above analysis, this paper proposes a bi-level optimization model for signal timing at expressway exit ramps and connecting intersections. The model focuses on exit ramps and their connecting intersections, omitting signal lights at the exit ramps, and addresses the problem of queue spillback to the mainline through signal timing at the connecting intersections. The maximum capacity of the approach lines in the direction of the exit ramp is set as the lower-level optimization objective, and the minimum average vehicle delay and the average number of stops per vehicle are set as the upper-level optimization objective, while taking into account the queue length in the direction of the exit ramp and other directions. By splitting the optimization objective into two layers, the trade-off between exit ramps and connecting intersections can be comprehensively considered. This approach helps to avoid excessively long red time for other directions, thereby preventing regional traffic congestion and providing a more optimized signal timing plan. Finally, we apply the particle swarm algorithm to solve the model and conduct simulation experiments on a real intersection using the VISSIM simulation software. The effectiveness of the model and algorithm is further validated through the SPSSAU platform.

## 2. Problem Description

**Figure 1:**
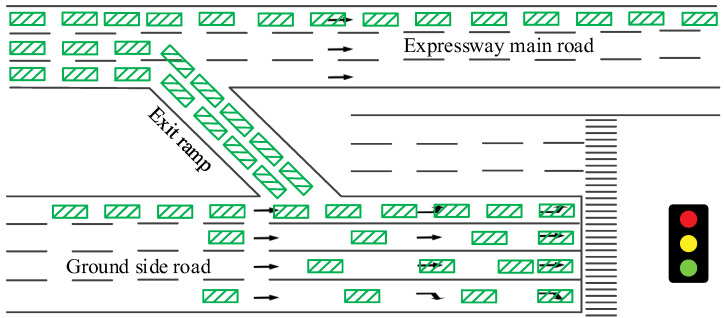
Schematic diagram of vehicle congestion in the exit ramp area.

During peak periods, traffic on the expressway is diverted toward the ground traffic flow at the exit ramp junction, which has a higher flow rate. If the vehicle evacuation capacity in this area is insufficient, it inevitably leads to traffic congestion. Additionally, the signal timing scheme at the exit ramp intersection may not release the green light in time according to the arrival flow of the approach line. This may result in queue overflow when the exit ramp articulation approach line flow is high and cannot be entirely released, causing the inability of vehicles on the expressway exit ramp to leave, even affecting the traffic on the mainline. Research [18] shows that adopting scientific signal control methods is an extremely effective measure to address such problems. Figure 1 shows the schematic diagram of vehicle congestion in the exit ramp area.

## 3. Bi-Level Programming Optimization Model Construction

In evaluating the performance of intersections, the average vehicle delay, the average number of stops per vehicle, and the capacity are widely used metrics. Generally, minimizing the average vehicle delay and the number of stops per vehicle have positive effects on improving the efficiency of traffic systems, but they focus on different perspectives and optimization objectives. The average vehicle delay primarily focuses on overall traffic smoothness and reducing overall delay time, while the number of stops per vehicle emphasizes reducing waiting time and improving travel continuity. When dealing with oversaturation at the exit ramp connecting to the approach lane during peak hours, a multi-objective optimization model that considers all of the metrics may struggle to strike a balance between the metrics. The advantages of the bi-level programming optimization model are that the optimization objectives are divided into upper and lower levels, making the objectives of each level clearer and better targeted.

By setting appropriate constraints, the bi-level programming optimization model can comprehensively consider the capacity of the exit ramp and the overall performance of the intersection, thereby identifying the optimal signal control strategy. Therefore, to ensure the capacity of the exit ramp, the maximum capacity of the approach lanes in the direction of the exit ramp is established as the lower-level optimization objective. However, solely considering the maximum capacity of the approach lanes in the direction of the exit ramp may affect the traffic flow of other approach lanes at the intersection. Thus, to improve the overall operation of the intersection, the average vehicle delay and the average number of stops per vehicle at the intersection are selected as the upper-level optimization objectives, and the queue length in each direction is taken as the main constraint so that the queue length in each direction will not overflow back to the mainline or upstream intersection.

### 3.1. Lower-Level Optimization Model

The lower-level optimization model takes the signal configuration parameters of the exit ramp articulation intersection as the optimization variables, with the maximum and minimum green time and the maximum queue length of the approach lanes of other phases as the constraints. The maximum capacity of the approach lanes in the direction of the exit ramp is set as the control objective to establish the objective function.

#### 3.1.1. Objective Function

At the end of the exit ramp, vehicles do not enter the articulated intersection approach lanes, and drivers can freely choose whether to change lanes. Therefore, the intertwining behavior of vehicles from different directions at the end of the exit ramp does not consider the discount factor on the intersection capacity. Let the number of phases be n, $g_{i}$ be the green time of the *i*-th phase, and, considering the variation in vehicle headway and number of lanes for different directions of the approach lanes, the design capacity of the approach lanes in the direction of the exit ramp be $Q^{ra}$. Considering the gap between the headway and the number of lanes in different directions of the entrance road, the expression of the control target is obtained by improving the formula of the parking section capacity [14]:

(1) $$Q^{ra}=\max\sum_{i}\alpha \frac{3600}{C}\left(\frac{g_{i}^{ra}-t_{0}}{h_{t}^{ra}}+1\right)m_{i}^{ra}$$

where C is the articulated intersection period (s); $g_{i}^{ra}$ is the green time for the *i*-th phase of the approach lanes in the direction of the exit ramp (s); $t_{0}$ is the time for the first vehicle passing the stop line after the green light (s), which, according to reference [19], is taken as 2.3 s; $h_{t}^{ra}$ is the headway of the *i*-th phase of the approach lines in the direction of the exit ramp passing the stop line (s) (the fleet of small cars in the straight lane is usually taken as 2.5 s, and the fleet of small cars in the left-turn lane is determined by actual situation); α is the discount factor, mainly reflecting the road environment interference by vehicles, taken as 0.9; and $m_{i}^{ra}$ is the number of lanes in *i*-th phase of the approach lanes of the exit ramp.

#### 3.1.2. Constraints

In intersection signal control, pedestrians must be considered to safely cross the street within the phase green time. The expression for the shortest green time for pedestrian crossing at an intersection [20] is:

(2) $$g_{min}=7+\frac{l_{i}}{v_{p}}-I$$

where $g_{min}$ is the minimum green time (s); $l_{i}$ is the pedestrian crossing length (m); $v_{p}$ is the pedestrian crossing speed (m/s), which can be 1.0 m/s; and I is the green interval time (s).

More green time should be assigned to the associated phase when considering the maximum capacity of the approach lanes in the direction of the exit ramp, so that the green time obtained by other phases is relatively reduced. In order to avoid the long queuing distance of the approach lanes of a phase, the approach queue length of a certain phase of the intersection is selected as the constraint condition of the objective function. Thus, the expression of the queue length $L_{ij}$ of the *j*-th approach of the *i*-th phase of the intersection [21] is:

(3) $$L_{ij}=\frac{q_{ij}(R_{i}-6)}{3600}\left[1+\frac{1}{S/q_{ij}-1}\right]\frac{h_{t}}{m_{ij}F_{m}}$$

where $q_{ij}$ is the actual traffic volume at the *j*-th approach lanes of the *i*-th phase (veh/h); $R_{i}$ is the red time at the i-th phase (s); S is the saturation flow (veh/h); $m_{ij}$ is the number of lanes in the *j*-th approach lanes of the *i*-th phase; $F_{m}$ is the lane utilization factor, taken as 0.75.

Add the green time of each phase to the green interval time to obtain the intersection signal cycle duration C:

(4) $$s.t.\left\{\begin{array}{c} C=\sum_{i=1}^{n}\left(g_{i}+I_{i}\right),i=1,2,\cdots ,n\\ g_{min}\leq g_{i}\\ L_{ij}\leq \theta \cdot L_{j} \end{array}\right.$$

where $I_{i}$ is the green interval time for the *i*-th phase of the approach lines in the exit ramp direction (s); θ is the maximum queue length reduction factor, taken as 0.9; $L_{j}$ is the distance from the stop line of the *j*-th approach lanes at the intersection to the exit lane of the upstream intersection (m).

### 3.2. Upper-Level Optimization Model

To ensure the overall efficiency of the articulated intersection, the upper-level optimization model establishes an objective function with the intersection signal period length; phase green time and road saturation as constraints; and the minimum average delay vehicle and the minimum number of stops per vehicle as the optimization objectives. This paper primarily focuses on the signal timing optimization at the intersection of the exit ramp articulation. If interweaving delay is considered, it will impact the saturation flow rate of the road and result in changes to the signal control schemes. Therefore, the stop line signal delay calculated by Webster’s formula is selected as the delay indicator. Additionally, considering the total delay resulting from both the weaving delay and signal delay of upstream vehicles is also an extension direction of our future work.

#### 3.2.1. Optimization Objectives

(1) The Average Vehicle Delay

As an important indicator to evaluate the operational efficiency of the articulated intersection, the average delay refers to the difference between the time required for vehicles to pass the intersection under normal driving conditions and obstructed conditions. Its magnitude visualizes the congestion and operational efficiency of the intersection. If the articulated intersection contains n signal phases, then the total delay time D is the sum of delays of each phase:

(5) $$D=\sum_{i=1}^{n}d_{i}q_{i}$$

where $d_{i}$ is the average vehicle delay of *i*-th phase (s); $q_{i}$ is the average traffic volume of *i*-th phase (veh/h).

Based on the Webster signal intersection delay formula [22], the expression of the average vehicle delay $d_{i}$ of *i*-th phase is obtained as:

(6) $$d_{i}=\sum_{j}\frac{C{\left(1-\lambda_{i}\right)}^{2}}{2(1-\lambda_{i}\cdot x_{ij})}+\sum_{j}\frac{x_{ij}{}^{2}}{2q_{ij}(1-x_{ij})}$$

where $\lambda_{i}$ is the green signal ratio of *i*-th phase, $\lambda_{i}=\frac{g_{i}}{C}$; $x_{ij}$ is the saturation of *j*-th approach lines of *i*-th phase, $x_{ij}=\frac{q_{ij}}{Q}$.

The average vehicle delay can be obtained by dividing the total vehicle delay during a signal cycle by the total number of vehicles passing through the intersection:

(7) $$\bar{d}=\frac{\sum_{i}d_{i}q_{i}}{\sum_{i}q_{i}}$$

(2) The Average Number of Stops Per Vehicle

The expression of the average number of stops per vehicle [11] of *i*-th phase is:

(8) $$h_{i}=\sum_{j}0.9\frac{1-\lambda_{i}}{1-\lambda_{i}x_{ij}}$$

Therefore, the average number of stops per vehicle can be obtained by dividing the total number of stops during one signal cycle by the total number of vehicles passing through the intersection:

(9) $$\bar{h}=\frac{\sum_{i}h_{i}q_{i}}{\sum_{i}q_{i}}$$

#### 3.2.2. Constraint Conditions

The constraints of the optimization objective function for the independent variables are mainly the following three:

(1) Phase green time constraints: Set the minimum green time and maximum green time;
(2) Signal cycle duration constraints: Set a minimum cycle duration and a maximum cycle duration;
(3) Road saturation constraint: The road saturation is relatively high during peak hours, set the minimum saturation to 0.7 and the maximum saturation to 0.9.

Therefore, the constraints expression is:

(10) $$s.t.\left\{\begin{array}{l} g_{min}\leq g_{i}\leq g_{max}\\ C_{min}\leq C\leq C_{max}\\ 0.7\leq x_{ij}\leq 0.9 \end{array}\right.$$

where $g_{i}$ is the phase green time (s); $g_{min}$ is the phase minimum green time (s); $g_{max}$ is the phase maximum green time (s); $C_{min}$ is the minimum signal cycle time (s); $C_{max}$ is the maximum signal cycle time (s).

#### 3.2.3. Objective Function

When considering the average vehicle delay and the average number of stops per vehicle as optimization objectives, since they have different dimensions, the average vehicle delay primarily focuses on overall traffic smoothness and reducing the overall delay, while the number of stops per vehicle emphasizes reducing parking wait time and improving travel continuity. In the process of signal optimization, more attention should be paid to the average vehicle delay during peak hours, while drivers during flat peak hours prefer to reduce the number of stops per vehicle. To comprehensively consider the preferences of these two objectives, this paper adopts a fuzzy compromise programming method to normalize the average vehicle delay and the average number of stops per vehicle. This method not only considers the degree to which each target is close to the ideal state, but also considers the degree of weight preference in the calculation process, which is more suitable for changing traffic conditions. It follows the approach outlined in reference [23].

(1) Calculate the ideal vectors, $X_{min}$ and $X_{max}$, consisting of minimum and maximum values for each objective expression under the constraints:

(11) $$X_{min}=(x_{1},x_{2})=({\bar{d}}_{\min},{\bar{h}}_{\min})$$

(12) $$X_{max}=(x_{1},x_{2})=({\bar{d}}_{\max},{\bar{h}}_{\max})$$

(2) Calculate the membership function expression of each objective function:

(13) $$U=\left\{\begin{array}{l} 1,X_{i}\leq X_{\min}\\ \frac{X_{\max}-X_{i}}{X_{\max}-X_{\min}},X_{\min}<X_{i}<X_{\max}\\ 0,X_{\max}\leq X_{i} \end{array}\right.$$

(3) Combine the fuzzy preference idea to calculate the weights.

Construct the preference matrix R as follows:

(14) $$R=\left\{\begin{array}{l} R(i,j)=0,R(j,i)=2(t_{i}<<t_{j})\\ R(i,j)=0,R(j,i)=1(t_{i}<t_{j})\\ R(i,j)=1,R(j,i)=1(t_{i}\approx t_{j}) \end{array}\right.$$

where $t_{i}$ denotes the degree of preference for the *i*-th target. When traffic congestion is serious, the average vehicle delay at the intersection should be given priority, and the average number of stops per vehicle frequency preference should be emphasized during flat peak hours. This paper assumes that $t_{2}<t_{1}$ is the peak period. The preference matrix of the two objectives is obtained as:

(15) $$R=\left(\begin{array}{cc} \begin{array}{l} 1\\ 0 \end{array} & \begin{array}{l} 1\\ 1 \end{array} \end{array}\right)$$

The preference relation matrix $R_{a}$ constructed from R is:

(16) $$R_{a}=\left\{\begin{array}{l} R_{a}(i,j)=\gamma ,R_{a}(j,i)=\gamma (R(i,j)=1\;\mathrm{and}\;R(j,i)=1)\\ R_{a}(i,j)=\alpha ,R_{a}(j,i)=\beta (R(i,j)=0\;\mathrm{and}\;R(j,i)=1)\\ R_{a}(i,j)=\beta ,R_{a}(j,i)=\alpha (R(i,j)=1\;\mathrm{and}\;R(j,i)=1) \end{array}\right.$$

Based on the preference relationship of $t_{2}<t_{1}$ and the preference demand for the average vehicle delay and the average number of stops per vehicle, the value taken here is α=0.25, β=0.75, γ=0.5. The preference relation matrix $R_{a}$ is obtained as:

(17) $$R_{a}=\left(\begin{array}{cc} \begin{array}{l} \gamma \\ \alpha \end{array} & \begin{array}{l} \beta \\ \gamma \end{array} \end{array}\right)=\left(\begin{array}{cc} \begin{array}{l} 0.5\\ 0.25 \end{array} & \begin{array}{l} 0.75\\ 0.5 \end{array} \end{array}\right)$$

The directed weighted graph G(A,R) is defined by the matrix $R_{a}$, and the boundary values of this graph is:

(18) $$S_{L}(a,R)=\sum {}_{C\in A\backslash \left\{a\right\}}R(a,c)$$

The weight factor is calculated as: $\lambda (t_{1})=\frac{S_{L}(t_{1},R)}{\sum {}_{t_{i}\in T}S_{L}(t_{i},R)}=\frac{0.75}{0.75+0.25}=\frac{3}{4}$

The same reasoning leads to: $\lambda (t_{2})=\frac{1}{4}$

(4) The converted single objective function f(x) is:

(19) $$f(x)=\max{\left[\sum_{i=1}^{3}{\left(\lambda_{i}u_{i}\right)}^{p}\right]}^{\frac{1}{p}}$$

where $\lambda_{i}$ is the weight of each objective function; p is the distance parameter.

Regarding the value of p, some scholars [24] found that, when p=+∞, the obtained signal timing parameters are optimal and the corresponding single objective function equation is obtained as:

(20) $$f(x)=\max\left[\min\sum_{i=1}^{3}\left(\lambda_{i}u_{i}\right)\right]$$

## 4. Model Solution

The bi-level programming optimization model constructed in this paper is a nonlinear optimization problem, and the particle swarm algorithm is characterized by its simple structure, fast convergence, and robustness in solving such global optimization problems [25].

The particle swarm optimization (PSO) algorithm first regards the possible solutions of the problem as particles, and secondly, the position of the particle in the search space is evaluated by the fitness value determined by the objective function, and the velocity of the particle is used to determine the direction and distance of the particle, and subsequently, the optimal particle is selected in the iteration to obtain the optimal solution in the solution space. The particle updates its velocity and position according to Equations (21) and (22):

(21) $$v(t+1)=\omega v(t)+c_{1}r_{1}(P_{i}-x(t))+c_{2}r_{2}(P_{g}-x(t))$$

(22) x(t+1)=x(t)+v(t+1)

where t is the current number of iterations; $c_{1}$ and $c_{2}$ are the learning factors; $r_{1}$ and $r_{2}$ are random numbers uniformly distributed between 0 and 1; and ω is the expression of the inertia coefficient as:

(23) $$\omega =\omega_{\max}-(\omega_{\max}-\omega_{\min})\times it/it_{\max}$$

where $\omega_{\max}$ and $\omega_{\min}$ are the maximum and minimum inertia coefficients; it and $it_{\max}$ are the current and maximum number of iterations.

The specific solution procedure is as follows:

Step 1: Randomly initialize the position $X_{i}$ and velocity $V_{i}$ of the particles in the particle swarm, i∈[1,m], where m is the population size. Set the maximum number of iterations and the upper and lower bounds, and the value range of the inertia coefficient ω. Create the initial solution that generates the lower-level optimization satisfying the constraints.

Step 2: Update the velocity and position of each particle in the population. Denote $P_{i}$ as the current position of the *i*-th particle and denote $P_{g}$ as the position of the best particle in the initial population.

Step 3: Substitute the position $X_{i}$ of the *i*-th particle in the upper-level optimization model into the lower-level optimization model to obtain the optimal solution $Y_{i}$ of the lower-level model.

Step 4: Substitute $X_{i}$ and $Y_{i}$ into the objective function of the upper-level optimization to calculate the fitness function value $F(X_{i},Y_{i})$ of the *i*-th particle, i∈[1,m].

Step 5: If the fitness of *i*-th particle is better than that of $P_{i}$, update $P_{i}$ to $X_{i}$. The optimal solution $yP_{i}$ of the lower-level model corresponding to $P_{i}$ is updated to $Y_{i}$. If the fitness of the *i*-th particle is better than that of $P_{g}$, $P_{g}$ is updated to $X_{i}$. The optimal solution $yP_{g}$ of the lower-level model corresponding to $P_{g}$ is updated to $Y_{i}$.

Step 6: If the algorithm reaches the maximum number of iterations, proceed to Step 8; otherwise, proceed to Step 7.

Step 7: Recalculate to obtain $P_{g}$, find the optimal solution $yP_{g}$ of the lower model corresponding to $P_{g}$, and return to Step 4.

Step 8: Output the optimal solutions $P_{g}$ and $yP_{g}$ of the bi-level optimization, obtain the objective function value of the bi-level optimization, and the algorithm ends.

## 5. Example Analysis

### 5.1. Basic Data

**Figure 2:**
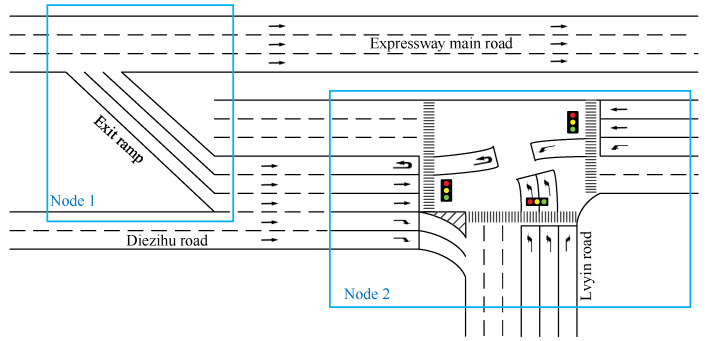
Schematic diagram of the channelization of an exit ramp connecting to an intersection area.

**Figure 3:**
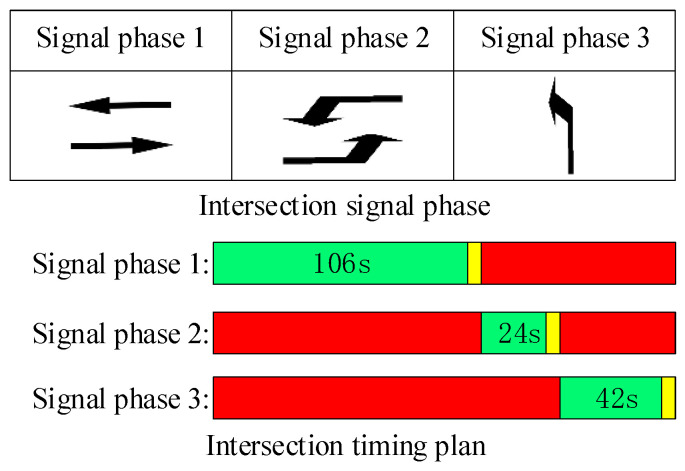
Current signal phase diagram and timing scheme of intersection.

T-intersection can provide a relatively simplified starting point for the preliminary verification and evaluation of model feasibility and performance. It can also serve as a benchmark test to compare and improve other intersection models. Additionally, in order to practically address the frequent congestion issue in the area of the T-intersection connecting the Huangjiahu Interchange exit ramp and the Diezihu Avenue in Nanchang city, this area is selected as an analysis example. The exit ramp area is set as Node 1 and the intersection area is set as Node 2. The drainage diagram of the intersection area connecting the exit ramp is shown in Figure 2, and the current signal phase diagram and timing scheme of the intersection are shown in Figure 3.

According to Figure 3, the signal timing scheme used at the intersection is a three-phase fixed signal timing scheme. Based on the field traffic survey and the calculation of the equivalent traffic volume, an observation period of 5 min is set to obtain the real traffic flow data at 5 min intervals. This paper focuses on the analysis of real traffic flow. The collected flow data are inherently random and time-varying. During peak hours, the traffic flow tends to exhibit continuity, and sudden changes are not expected under normal circumstances. Hence, we utilize the 5 min flow data from each approach lane as input to determine the signal control timing for the subsequent 5 min. Table 1 shows the continuous 5 min traffic statistics for each approach lane’s flow direction and expressway exit ramp at the intersection during the morning peak hours (7:30–9:00) on a weekday (Monday, 12 October 2021).

The number of lanes in the eastern, western, and southern approach lanes of the intersection area are 3, 5, and 3, respectively; the pedestrian crossing lengths are 23 m, 33 m, and 20 m, respectively; and the distances from the upstream intersection (or expressway) are 325 m, 138 m, and 373 m, respectively.

After the calculation and review of the related literature [26], the minimum green time for the first phase of the signal intersection is 24 s, the minimum green time for the third phase is 27 s, the maximum green time $g_{\max}$ is 90 s, the green time interval is 3 s, the minimum cycle duration $C_{\min}$ is 72 s, and the maximum cycle duration $C_{\max}$ is 180 s. The saturation flow rate is taken as 1900 pcu/h with reference to the literature [20].

### 5.2. Model Application

The particle swarm algorithm is implemented using MATLAB R2018. After multiple simulation experiments for optimization and debugging, the optimal parameter settings are selected. The parameter settings are as follows: the population size *m* is 50, the maximum number of iterations *t* is 100, the learning factor for $c_{1}$ and $c_{2}$ is 2, the maximum value of the inertia coefficient $\omega_{\max}$ is 0.9, and the minimum value $\omega_{\min}$ is 0.1. The independent variables of the objective function are the green time and the signal cycle duration of each phase, the initial speed and position are generated randomly, and the algorithm terminates when the maximum number of iterations is reached. According to the 5 min update period, the optimal signal timing scheme in each time period is calculated as shown in Table 2.

### 5.3. Simulation Comparison Analysis

The traffic simulation software VISSIM is used to establish the simulation scenario [27]. Under the same road network conditions and traffic flow conditions, the dynamic simulation of the signal timing scheme before and after the optimization is carried out, mainly to compare and evaluate the optimization degree of the average vehicle delay and the average number of stops per average and the queue overflow situation between Node 1 and Node 2 under different timing schemes; the simulation duration is set to 3600 s. To test the effectiveness of our model, we conducted comparisons with the following three methods:

(1) Fixed-Time Control Method: Traffic lights follow a fixed sequence cycle. The duration of each phase is pre-set and remains unchanged regardless of traffic flow. The fixed signal timing scheme is the timing scheme for the current situation of the intersection we investigated, as shown in Figure 3.
(2) Webster Control Method: This method determines the optimal signal cycle length based solely on minimizing the total vehicle delay, and subsequently determines the timing scheme accordingly.
(3) Multi-Objective Control Method: A mathematical model was established, considering constraints such as minimizing the average vehicle delay and the average number of stops per vehicle, maximizing the overall intersection capacity, and ensuring effective green signal time for each phase and intersection cycle length, as proposed by Xu et al. [17].

**Figure 4:**
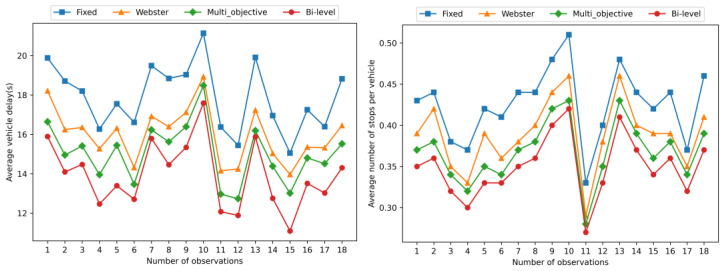
Data of the simulation results of the signal timing scheme before and after the optimization method at Node 1.

**Figure 5:**
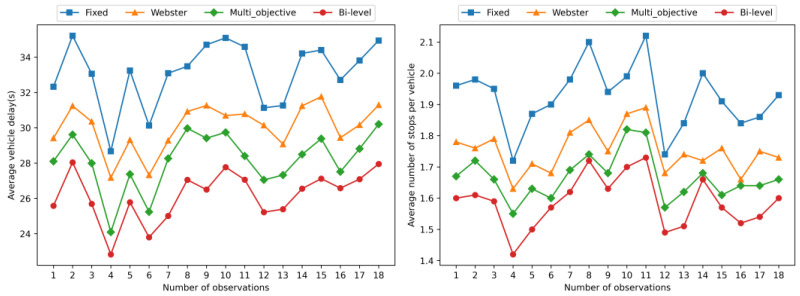
Data of the simulation results of the signal timing scheme before and after the optimization method at Node 2.

The comparison data related to the vehicle traffic condition in the road after the simulation are obtained as shown in Figure 4 and Figure 5.

As shown in Figure 4, it is evident that both Webster control method and multi-objective control method have effectively reduced the average vehicle delays and the average number of stops per vehicle in the exit ramp and mainline areas compared to the original fixed-time control method. Furthermore, additional data demonstrate that our proposed bi-level optimization control method exhibits even more significant improvements, significantly outperforming the other three methods. This is due to the fact that we focus on maximizing the traffic capacity of the exit ramp direction, which consequently minimizes vehicle delays and stops, effectively alleviating congestion spillover from the exit ramp to the mainline.

In addition to optimizing the capacity of the exit ramp, our method takes into full consideration the overall efficiency of the intersection. The results in Figure 5 demonstrate that our proposed method significantly outperforms the other three methods in the converging intersection area. By comprehensively considering the queuing situations in all directions, we have successfully enhanced the overall traffic capacity of the intersection, reducing vehicle delays and the number of stops, thereby effectively improving traffic fluency in the intersection area.

Table 3 summarizes the average vehicle delay and average number of stops per vehicle for the four methods, as follows:

In the exit ramp area of Node 1, compared to the original fixed-time control method, the Webster control method, multi-objective control method, and proposed bi-level optimization method achieved average vehicle delay reductions from 17.88 s to 15.99 s, 15.04 s, and 13.93 s, respectively, showing reductions of 10.57%, 15.88%, and 22.09%. The average number of stops per vehicle decreased to 0.39, 0.37, and 0.35, respectively, compared to the current value of 0.43, representing reductions of 9.30%, 13.95%, and 18.60%.

In the intersection area of Node 2, the average vehicle delay decreased to 30.05 s, 28.16 s, and 26.17 s, respectively, showing reductions of 9.24%, 14.95%, and 20.96%, compared to the original delay of 33.11 s. The average number of stops per vehicle decreased to 1.75, 1.66, and 1.59, respectively, compared to the current value of 1.92, resulting in reductions of 8.85%, 13.54%, and 17.19%.

In summary, both the Webster method and the multi-objective method demonstrated better performance than the original fixed-time signal control in the exit ramp area and intersection area. However, the proposed bi-level optimization method outperformed the other two methods in terms of its effectiveness.

The paired *t*-test can assess the practical effectiveness of different methods and judge whether the experimental results have practical significance. With the assistance of the SPSSAU platform, we conducted a paired *t*-test analysis on the simulated data of the proposed optimization method and the other three methods. The test results are shown in Table 4.

According to Table 4, it can be observed that our proposed method and the other three methods show significant differences at the 0.01 significance level (P=0.000). The Cohen’s *d* value in the table represents the effect size (magnitude of differences), where values of 0.20, 0.50, and 0.80 are used as thresholds to differentiate small, medium, and large effect sizes, respectively. The larger the value, the greater the differences between the methods. Our proposed method shows Cohen’s *d* values greater than 0.8, indicating substantial differences compared to the other three methods. The results of the paired *t*-test further support the significant differences between the simulation results of our proposed signal optimization method and the other three methods, providing additional evidence of the improvement in the signal optimization method. Without altering road infrastructure and vehicle conditions, our optimized signal timing scheme successfully enhances the traffic capacity of both exit ramps and connecting intersection. As a result, the effectiveness of our model and algorithm is validated.

## 6. Conclusions

By optimizing the signal timing scheme of the articulated intersection of the expressway exit ramp to improve the overall operation quality and prevent the long queue in the exit ramp, the author proposes a signal timing bi-level programming optimization model. The model optimization result finds a balance between the expressway exit ramp and the articulated intersection, which not only realizes the exit ramp vehicles “coming out”, but also ensures the overall good operation of the articulated intersection. The signal parameters that are better than the current situation are solved by the particle swarm optimization algorithm. Finally, the VISSIM and SPSSAU platforms are used to simulate and compare. Through the difference test, it further shows that the optimization method established in this paper has a good improvement effect on the traffic congestion problem of the researched expressway exit ramp articulated intersection area, and provides a certain reference to improve the service level of urban expressway exit ramp.

At present, the model only considers the signal timing optimization of the expressway exit ramp articulation intersection, and the next step would be to consider the entrance ramp, upstream intersections, and interwoven delays, and conduct research on signal optimization for the overall integration of exit ramp, entrance ramps, and connecting intersections.

## Figures and Tables

**Table 1:** Statistical traffic volume (veh) for consecutive 5 min during peak hours.

Number of Observations	West Entrance Road			East Entrance Road		South Entrance Road		Exit Ramp
	Turn Left	Straight	Turn Right	Turn Left	Straight	Turn Left	Turn Right	
1	38	120	79	18	130	67	14	171
2	53	128	73	18	134	46	10	177
3	40	115	83	22	127	61	11	171
4	32	107	78	22	102	46	13	156
5	38	98	74	26	98	64	16	150
6	29	114	79	21	122	53	11	161
7	50	138	88	16	158	35	11	189
8	41	104	72	17	112	67	13	156
9	42	101	67	17	126	67	12	151
10	43	112	60	21	122	55	16	155
11	40	128	73	16	127	37	14	174
12	35	109	65	20	119	61	14	157
13	37	115	77	21	127	56	11	165
14	40	108	67	18	113	53	13	155
15	42	104	62	22	109	50	17	153
16	36	110	68	18	120	49	14	155
17	40	107	60	18	112	47	13	149
18	42	109	67	21	113	53	12	157

**Table 2:** Optimal signal timing for each phase of a five-minute update cycle.

Number of Observations	Phase Green Light Duration/s			Signal Period Duration/s
	First Phase	Second Phase	Third Phase	
1	86	28	31	154
2	90	28	35	162
3	84	29	29	151
4	81	24	26	140
5	83	27	30	149
6	82	23	26	140
7	90	31	33	163
8	80	31	30	150
9	78	30	32	149
10	84	29	30	152
11	87	24	30	150
12	86	28	31	154
13	84	23	31	147
14	87	24	30	150
15	88	22	33	152
16	87	24	30	150
17	83	23	32	147
18	87	24	30	150

**Table 3:** Network performance with different control methods.

Area	Performance Index	Fixed-Time	Webster	Multi-Objective	Proposed Method
Node 1	Delay(s)	17.88	15.99 (−10.57%)	15.04 (−15.88%)	13.93 (−22.09%)
	Stop times	0.43	0.39 (−9.30%)	0.37 (−13.95%)	0.35 (−18.60%)
Node 2	Delay(s)	33.11	30.05 (−9.24%)	28.16 (−14.95%)	26.17 (−20.96%)
	Stop times	1.92	1.75 (−8.85%)	1.66 (−13.54%)	1.59 (−17.19%)

**Table 4:** Paired *t*-test effect size indicator.

Method	Node	Performance	Mean Difference	95%CI	t	P	Cohen’s d
Fixed-time —Bi-level	Node 1	Delay	3.957	3.783–4.132	47.895	0.000	11.289
		Stop times	0.076	0.070–0.082	28.208	0.000	6.647
	Node 2	Delay	6.943	6.575–7.311	39.843	0.000	9.391
		Stop times	0.336	0.319–0.353	41.132	0.000	9.882
Webster —Bi-level	Node 1	Delay	2.062	1.806–2.317	17.019	0	4.012
		Stop times	0.039	0.033–0.045	14.577	0	3.438
	Node 2	Delay	3.879	3.543–4.214	24.387	0	5.748
		Stop times	0.166	0.143–0.188	15.757	0	3.737
Multi-objective —Bi-level	Node 1	Delay	1.111	0.878–1.344	10.063	0.000	2.372
		Stop times	0.018	0.016–0.020	20.283	0.000	4.781
	Node 2	Delay	1.9 96	1.685–2.307	13.544	0.000	3.194
		Stop times	0.076	0.058–0.093	9.011	0.000	2.124

## Data Availability

The data used to support the research of this paper are available from the corresponding author upon reasonable request.
